# Microsatellite markers for the *Jaera albifrons* species complex (marine isopods)

**DOI:** 10.1186/s13104-015-1595-9

**Published:** 2015-11-02

**Authors:** Ambre Ribardière, Thomas Broquet, Claire Daguin-Thiébaut

**Affiliations:** CNRS, Station Biologique de Roscoff, Sorbonne Universités, UPMC Univ Paris 06, UMR 7144, Team Diversity and Connectivity of Coastal Marine Landscapes, 29680 Roscoff, France

**Keywords:** SSR, Species complex, Cross-amplification, 454 pyrosequencing, Multiplex PCR

## Abstract

**Background:**

The *Jaera albifrons* complex contains five species of marine isopods (*J. albifrons*, *J. praehirsuta*, *J. ischiosetosa*, *J. forsmani*, and *J. posthirsuta*). These species, occurring on the shores of the North-Atlantic Ocean, are partially reproductively isolated by barriers due to sexual isolation (mate choice), genetic incompatibilities, and ecological specialization. Microsatellite loci would be useful for parentage-based analyses of sexual selection and studies of genetic structure in the context of speciation.

**Findings:**

Twenty-four microsatellite markers were developed for *J. albifrons* using pyrosequencing of enriched libraries. Patterns of polymorphisms were analyzed in 49 *J. albifrons* adult males sampled in two populations from Brittany (Western France). The average number of alleles per locus was 4.73 ± 2.45 and the average gene diversity was 0.55 ± 0.23. Most markers also successfully amplified in the three sibling species *J. praehirsuta*, *J. ischiosetosa*, and *J. forsmani*.

**Conclusions:**

These polymorphic and cross-amplifiable markers will be useful for population genetics and parentage studies in the *J albifrons* complex.

## Findings

### Background

The *Jaera albifrons* complex (Leach, 1814) is composed of five intertidal isopod species [1, 2]. Three species (*J. albifrons*, *J. praehirsuta*, and *J. ischiosetosa*) have a large distribution along the coasts of the North-Atlantic Ocean from South-Spain and South-USA up to Baltic and Arctic regions, while the two other species are restricted either to the North-American East coast (*J. posthirsuta*) or the European coasts (*J. forsmani*). Interest in these species stems from the fact that gene flow is interrupted by several isolating barriers but hybridization can occur under particular circumstances. Isolating barriers include ecological isolation, sexual isolation, and genetic incompatibilities [3, 4]. Microsatellite loci will be useful for population genetic studies of the *J. albifrons* complex, and fine scale analyses requiring parentage assignment (e.g. for investigating mechanisms of sexual selection). Microsatellite markers were developed using two French *J. albifrons* populations and cross-amplification was tested for the three other species that are found in Europe (*J. praehirsuta*, *J. ischiosetosa*, and *J. forsmani*).

### Methods

Total genomic DNA was isolated from seven *J. albifrons* individuals (three males and four females) using the Nucleospin^®^ Tissue kit (Macherey–Nagel) and sent to Genoscreen (Lille, France) for microsatellite development. Libraries enriched for microsatellites were prepared according to Malausa et al. [5] and sequenced on a 454 GS-FLX Titanium pyrosequencer (Roche). Among 42,661 raw sequences, 2609 microsatellite motifs were detected using QDD v1 [6] with default parameters, yielding 168 potential primer pairs. Among them, 95 primer pairs maximizing the number of repeats were tested for amplification and polymorphism using a set of seven *J. albifrons* individuals. Nine loci were found to be monomorphic, 46 loci did not yield amplification products, and 16 gave uninterpretable amplification patterns. The remaining 24 promising loci included dinucleotide, trinucleotide and tetranucleotide repeat motifs (Table 1).

**Table 1:** Microsatellite loci for *Jaera albifrons* and multiplex PCR conditions

Locus	F primer seq. (5′ to 3′)	R primer seq. (5′ to 3′)	Motif	Range size (pb)	GenBank Accession no.	PCR multiplex	Dye	Primer (10 µM)			Init. temp.	Fin. temp.	No. final cycles
								F** (µl)	F (µl)	R (µl)			
Ja30	CGTCATTTATGCGTGCGGTA	CGTCCATTCTTCATCTGACTGC	(TC)_9_	173–185	KP749881	1	PET	0.1	0.2	0.3	60	50	20
Ja37	TCGAGCATCATACGACGACA	GCCTATAGTGTCTTTGGCACC	(GA)_8_	101–115	KP749883		FAM	0.4	0	0.4			
Ja41	GGTGTTGGCGGAAAGATTCT	AGCCTACTCTACCGTCTGTT	(GA)_8_	172–180	KP749885		NED	0.1	0.2	0.3			
Ja91	ATCGGCATTCCACTGAGAGG	CCAGTCTTGAATGCCTTGGG	(CCT)_6_	073–082	KP749896		VIC	0.1	0.2	0.3			
Ja01	CAATCTTCGTCTTGGCGTCT	CACTGTGGGCTTAGGATTGT	(AG)_8_	226–247	KP749875	2	FAM	0.5	0	0.5	57	47	20
Ja02	TGCCACCAATTAACCAAACA	CGACAGCCATAATGATCACC	(CT)_10_N_16_(CT)_5_	182-212	KP749876		NED	0.1	0.2	0.3			
Ja35	TGGATTCACATTGCTTCCTGG	TCACGCATTCTAGTCTACGCT	(AT)_8_	173–179	KP749882		VIC	0.3	0.1	0.4			
Ja47	GGTGCTTAATGTAGTAGAGCGG	ATGGAACCACAAAACGGACG	(TG)_10_	116–124	KP749886	3	NED	0.1	0.2	0.3	60	50	20
Ja56	ACTAACACAAACATGCACTTACA	ATTGTTAGGTGCCTGCCATT	(ACA)_6_	261–276	KP749888		FAM	0.3	0.3	0.6			
Ja58	CCCACTGGACCACTACTTGA	ACCCTATCGTTGTAGTTGAGGT	(CT)_5_	186–188	KP749889		VIC	0.3	0.1	0.4			
Ja99	ATCAAACATGCAGCGGTGTC	AGGCATCTCAGAGGAATCACTT	(TC)_5_	152–154	KP749898		PET	0.1	0.2	0.3			
Ja21	TTCAAATTAAATGCAACGATG	CATTATTCTGAAGCTGTTGGTC	(GA)_10_	272–286	KP749878	4	NED	0.25	0	0.25	60	50	25
Ja71	CTGCCCTATCAGTTGAGCTT	CACAGTGCCATCAACAAAGC	(GA)_7_	263–269	KP749892		VIC	0.3	0	0.3			
Ja78	CACTCTACAGCAGCATAAGAGT	AGAACAAGGCAAGATGAGCTC	(AC)_8_	243–254	KP749893		PET	0.2	0.1	0.3			
Ja94	TGGTCCGATCGTGAGTTTCA	CCTTCGGATGGTAATAGGGCA	(AT)_6_	101–111	KP749897		PET	0.1	0.2	0.3			
Ja22	GCAAACTCGATCACCATTGGA	ACCCACCGTCGTCTATCAAC	(CT)_9_	106–128	KP749879	5	VIC	0.2	0.1	0.3	60	50	23
Ja64	TAAGCCGAGTCTCAACAGCA	CCCGTGCATAGCGAACAG	(TG)_5_N_22_(TG)_5_	119–127	KP749890		PET	0.1	0.2	0.3			
Ja66	CGCAAGTACAAATTTCTCTATGCT	TCGAACGGTACTCAATGTGAAG	(TAGA)_5_	175–187	KP749891		FAM	0.4	0	0.4			
Ja80	TGATGGATGAGAGGTGATCGT	TTGATTCAGATCCGATACCATGT	(GA)_6_	154–154	KP749894		NED	0.2	0.1	0.3			
Ja13	ATCCTTCATGAGTCCCGAGT	AAGTATGTCCGAACTACCGC	(GA)_12_	211–233	KP749877	6	VIC	0.2	0.1	0.3	57	47	23
Ja23	CAGGCCATCTTGCTGCAGAT	ACAGCCTCTCCATCATGCGT	(GA)_9_	120–134	KP749880		VIC	0.1	0.2	0.3			
Ja39	CGTGCAGTCATCTCAGTCAG	GCTGAGGAGAGCGAGTAATC	(TC)_8_	128–144	KP749884		NED	0.15	0.15	0.3			
Ja55	ACAACAGCAACAACTTCCGT	AGTTGTGATGTGGAGGAGCA	(CAA)_6_	130–138	KP749887		PET	0.15	0.15	0.3			
Ja82	TCCGAATGCACCAAATCTGA	GGCTTGTATTCGAATTTACATGGA	(GAA)_6_	292–295	KP749895		FAM	0.4	0	0.4			

The volume of fluorescent (F**) and non-fluorescent (F) forward primer was adjusted for each marker. Columns Init. temp. and Fin. temp. give the initial and final temperatures (in  °C) used for the touchdown PCR. Amplification products from multiplex 4–6 were diluted before electrophoresis (dilution level: 1/3)

Polymorphisms of these 24 loci were estimated in two populations of *J. albifrons* from Brittany: Lingoz (48°39′12.31ʺN, 3°57ʹ0.43ʺW, *n* = 24 males) and Inizan (48°39′34.09″N, 3°56′25.66″W, *n* = 25 males), for which genomic DNA was extracted from entire individuals using NucleoSpin^®^ 96 Tissue kit (Macherey–Nagel). Locus amplification was performed in six multiplex PCRs (three to five loci per PCR, Table 1), in 15 µl solutions containing 13 μl of reaction mixture and 2 µl of template DNA. Reaction mixtures contained 0.5 U of Gotaq G2 Hotstart DNA polymerase (Promega), 1× PCR buffer, 0.25 mM of each dNTP, 2 mM of MgCl_2_, 0.1 mg/ml of bovine serum albumin, and primers in locus-specific concentrations (Table 1). We used a touchdown PCR method, performed by a T100 Thermal Cycler (Bio-RAD) with the following conditions: initial denaturation at 95 °C for 4 min, followed by ten cycles of 95 °C for 30 s, annealing for 30 s with temperature step-downs (1 °C at each cycle) starting at an initial temperature specific to each multiplex (Init. temp. in Table 1), and 72 °C for 30 s. This was followed by 20–25 final cycles of 95 °C for 30 s, final temperature (Fin. temp.) for 30 s, 72 °C for 30 s, and a final elongation at 72 °C for 10 min.

PCR products were electrophoresed in a ABI 3130XL capillary sequencer (Applied Biosystems) together with the SM594 size marker [7] and electropherograms were analyzed using Genemapper v4 (Applied Biosystems). The number of alleles per locus, allelic richness, and observed and expected heterozygosity were estimated in Fstat vs. 2.9.3.2 [8]. This software was also used to test for Hardy–Weinberg equilibrium (Global test, option “HW within samples”, 10,000 permutations, Bonferroni correction applied), population differentiation, and linkage disequilibrium (option “between all pairs of loci in each sample”, 11,040 permutations). The presence of null alleles was tested using Micro-Checker vs. 2.2.3 [9].

Finally, the transferability of these markers was tested on three other species from the *J. albifrons* complex: *J. praehirsuta* (*n* = 74 males from five European populations), *J. ischiosetosa* (*n* = 18 males from two North-American populations), and *J. forsmani* (*n* = 8 males from one European population).

### Results and discussion

The average number of alleles per locus for the two pooled *J. albifrons* populations (n = 49) was 4.73 ± 2.45 and the average gene diversity was 0.55 ± 0.23 (details per locus and population in Table 2). All loci were polymorphic (2–13 alleles per locus) except Ja80, which is nonetheless reported here because it was polymorphic in *J. praehirsuta* and *J. forsmani* (Table 2) and could thus be useful at least for these species. Microsatellites Ja01, Ja55, and Ja64 deviated significantly from HWE (*p* < 0.001 in one of the two populations). Micro-checker results suggested that null alleles might be segregating at these loci as well as three additional markers associated with large F_IS_ values (Ja13, Ja27, and Ja58). Null alleles are often unavoidable in highly polymorphic species such as many marine invertebrates [10] and relevant microsatellite loci should be used only in analyses where their effect can be detected and corrected (e.g. parentage assignment). Moreover, the occurrence of null alleles is expected to be variable across geographic regions and species, so that the results reported here for two populations might not apply to other areas or species (our two samples came from nearby, albeit differentiated populations, F_ST_ = 0.01, *p* < 0.0001). Markers used in empirical studies should be chosen accordingly, and the multiplex design proposed here could be adapted. There was no linkage disequilibrium for any pair of loci.

**Table 2:** Polymorphism of *Jaera albifrons* microsatellite loci

Locus	Lingoz (*n* = 24)				Inizan (*n* = 25)				Cross-amplification (no. alleles)		
	No. alleles	Ho	He	F_IS_	No. alleles	Ho	He	F_IS_	*J. praehirsuta* (n = 74)	*J. ischiosetosa* (*n* = 18)	*J. forsmani* (*n* = 8)
Ja30	4	0.63	0.64	0.031	6	0.72	0.71	−0.013	7	5	6
Ja37	4	0.39	0.67	0.418	6	0.32	0.56	0.432	9^#^	5^#^	4
Ja41	4	0.46	0.45	−0.016	5	0.56	0.50	−0.113	10	8	5
Ja91	4	0.50	0.53	0.053	3	0.60	0.56	−0.137	6	3	4
Ja01	9	0.43	0.85	0.496*	11	0.59	0.84	0.302	22	3^#^	4^#^
Ja02	7	0.71	0.73	0.035	6	0.76	0.68	−0.118	11	4	5
Ja35	5	0.54	0.58	0.074	4	0.64	0.66	0.035	7	2	4
Ja47	4	0.67	0.61	−0.087	4	0.40	0.55	0.278	5	1	4
Ja56	6	0.67	0.62	−0.071	4	0.28	0.35	0.200	8	4	3
Ja58	2	0.00	0.08	1.000	2	0.08	0.27	0.713	6	2	1
Ja99	2	0.38	0.31	−0.211	2	0.36	0.35	−0.029	3	2	2
Ja21	5	0.57	0.69	0.183	6	0.52	0.70	0.257	12	6	3
Ja71	4	0.68	0.76	0.101	5	0.72	0.71	−0.013	12	9	−^#^
Ja78	5	0.61	0.75	0.197	5	0.71	0.70	−0.009	8	6^#^	4^#^
Ja94	5	0.63	0.68	0.078	5	0.56	0.70	0.198	9	5	5
Ja22	6	0.79	0.73	−0.094	6	0.52	0.58	0.102	10	6	2
Ja64	2	0.04	0.31	0.869*	2	0.12	0.35	0.662	6	2	3
Ja66	3	0.25	0.29	0.140	2	0.16	0.15	−0.067	5	1#	1
Ja80	1	NA	NA	NA	1	NA	NA	NA	4	1	2
Ja13	9	0.54	0.71	0.240	8	0.40	0.60	0.332	16	8	10
Ja23	4	0.50	0.69	0.275	7	0.76	0.71	−0.078	8	5	4
Ja39	8	0.92	0.85	−0.077	12	0.88	0.89	0.008	15	2	5^#^
Ja55	3	0.17	0.59	0.720*	5	0.48	0.67	0.289	9	3	4
Ja82	2	0.29	0.25	−0.150	2	0.12	0.12	−0.043	2	2	2

The number of alleles, expected heterozygosity (He), observed heterozygosity (Ho) and F_IS_ are given for each locus in two populations of the *J. albifrons* species (* indicates a significant deviation from HWE, *p* < 0.05). The number of alleles obtained through cross-amplification is given for samples of *J. praehirsuta*, *J. ischiosetosa*, and *J. forsmani* (^#^ indicates amplification success below 85 %, see text). Loci presented in the same order as in Table 1

Cross-species amplification was considered successful if more than 85 % of the individuals tested produced a good quality genotype at the first attempt and without optimization. With this criterion, 23 out of 24 microsatellite markers successfully amplified in *J. praehirsuta* (*n* = 74), 20 in *J. ischiosetosa* (*n* = 18) and 20 in *J. forsmani* (*n* = 8). These loci appeared to be polymorphic in nearly all cases (Table 2). These markers seem readily transferable to other species for European populations of *J. praehirsuta* and *J. forsmani*, and even to North-American populations in the case of *J. ischiosetosa*. The panel of microsatellites reported here thus provides a useful set of markers for parentage analyses and studies of the interspecific genetic structure within the *J. albifrons* complex.

## Availability of supporting data

The sequences containing microsatellite motifs are available through the National Centre for Biotechnology Information under accession numbers KP749875 to KP749898 (http://www.ncbi.nlm.nih.gov/).
